# Mitosis, Focus on Calcium

**DOI:** 10.3389/fphys.2022.951979

**Published:** 2022-06-17

**Authors:** Charlotte Nugues, Nordine Helassa, Lee P. Haynes

**Affiliations:** ^1^ Department of Molecular Physiology and Cell Signalling, Institute of Systems, Molecular and Integrative Biology, University of Liverpool, Liverpool, United Kingdom; ^2^ Department of Cardiovascular and Metabolic Medicine, Institute of Life Course and Medical Sciences, Faculty of Health and Life Sciences, University of Liverpool, Liverpool, United Kingdom

**Keywords:** calcium, mitosis, GCaMP Ca2+ imaging, regulation, cytokinesis

## Abstract

The transformation of a single fertilised egg into an adult human consisting of tens of trillions of highly diverse cell types is a marvel of biology. The expansion is largely achieved by cell duplication through the process of mitosis. Mitosis is essential for normal growth, development, and tissue repair and is one of the most tightly regulated biological processes studied. This regulation is designed to ensure accurate segregation of chromosomes into each new daughter cell since errors in this process can lead to genetic imbalances, aneuploidy, that can lead to diseases including cancer. Understanding how mitosis operates and the molecular mechanisms that ensure its fidelity are therefore not only of significant intellectual value but provide unique insights into disease pathology. The purpose of this review is to revisit historical evidence that mitosis can be influenced by the ubiquitous second messenger calcium and to discuss this in the context of new findings revealing exciting new information about its role in cell division.

## Introduction

Since the discovery of the cyclins and their involvement in the cell-cycle, the study of mitosis regulation has focused on coordinated patterns of protein synthesis, phosphorylation, de-phosphorylation, and degradation (Johnson and Walker, 1999; Nurse, 2002; McIntosh, 2016). Study of cyclin biology has demonstrated their pivotal importance during the cell cycle and mitotic cell division and, as noted by Whitaker and Larman (Whitaker and Larman, 2001), this system, in principle, does not require secondary messengers. However, cell signalling is often more complex than initially appears and for over 50 years, calcium biology researchers have suspected this multifunctional ion plays an important part in the control of mitosis (Perris and Whitfield, 1967; Luckasen et al., 1974; Berridge, 1975).

### Plant and Animal Cells

These ideas were founded on early studies employing model plant (Saunders and Hepler, 1982; Hepler and Callaham, 1987; Hepler, 1994) and animal systems (Poenie et al., 1985; Silver, 1990; Whitaker, 2006a; B. Silver, 1989; Silver, 1986; Silver, 1996) where mitotic calcium signals were observed with chemical dyes (Poenie et al., 1985) or protein-based sensors of calcium such as aequorin (Keating et al., 1994). Model systems were chosen for their experimental tractability and robustness. They are often large cells and therefore easy to manipulate (microinjection, dye loading, pharmacological intervention), with minimal perturbation to the mitotic apparatus (Sluder et al., 1998; Sluder, 2016). Such studies provided consistent and promising results to link calcium with mitosis and highlighted significant correlations between calcium signals and mitotic landmarks such as nuclear envelope breakdown (NEB) (Poenie et al., 1985; Browne et al., 1996) and the metaphase → anaphase transition (Poenie et al., 1985; Hepler and Callaham, 1987) (when the spindle retracts and pulls sister chromatids towards opposite poles of what will become each new cell). Various studies elaborated on these experimental approaches to disrupt mitotic calcium signals through the introduction (microinjection and later, by use of cell-permeant AM esterified variants) of calcium buffers such as BAPTA and showed that this inhibited mitosis progression (Miller et al., 1993). This inhibition could be relieved by addition of excess free calcium, completing the circle, and demonstrating a fundamental role for calcium in cell division (Steinhardt and Alderton, 1988; Groigno and Whitaker, 1998). Further evidence that strengthened a role for calcium during mitosis came from related work where inositol 1,4,5 trisphosphate (IP_3_) signalling was observed (Ciapa et al., 1994) and then manipulated with drugs such as heparin or introduction of antibodies directed towards the parent lipid, phosphatidylinositol 4,5 bisphosphate. These approaches were intended to suppress endoplasmic reticulum (ER) calcium release mediated by IP_3_ receptors (IP_3_Rs) and they altered the kinetics of mitosis (Han et al., 1992; Ciapa et al., 1994). Conversely, elevation of cytoplasmic IP_3_ prematurely activated mitosis in sea urchin eggs (Whitaker and Irvine, 1984; Twigg et al., 1988). These findings indicated an important role for calcium during mitosis and highlighted the ER as the likely calcium store. They were also consistent with independently derived biochemical properties of key structures such as the mitotic spindle, that is, its disassembly in response to calcium, leading to the logical speculation that one major target of calcium during mitosis are spindle microtubules. A simple interpretation of these data would be that disruption of calcium signalling in cells, the source of which could be the ER (based on IP_3_ related experiments), prevents mitosis progression through a mechanism whereby the spindle is stabilised and cannot retract as normal. Exactly how calcium is thought to modify microtubules along with more recent data in this specific area of research will be discussed in greater detail later.

### Mammalian Cells

A natural extension of research into the role of calcium during mitosis was to look for corresponding signals in mammalian cells. Most of this work, but not all (Swierenga et al., 1976), employed cultured cell lines, presumably due to: 1) Ease of use and availability; 2) Technical barriers to culturing and manipulating primary cells and 3) Cultured cell lines being transformed, dividing rapidly, therefore well-suited to the study of mitosis. There are many excellent reviews that cover the work on mammalian cells, in detail, including those of Whitaker (Whitaker and Larman, 2001; Whitaker, 2006b), Hepler (Hepler, 1994), Silver (Silver, 1990; Silver, 1996) and Santella (Santella, 1998) therefore it will only be summarised here. Manipulation of calcium levels was shown to impact on mitosis (Izant, 1983) and calcium signals were observed and correlated, as for other model systems, with mitotic processes such as NEB and metaphase → anaphase transition (Poenie et al., 1986; Ratan et al., 1986). Frustratingly however there were studies, often using the same cell lines and sometimes from the same laboratories, that provided conflicting data (Ratan et al., 1986; Tombes and Borisy, 1989; Kao et al., 1990). Either calcium signals were observed but there was no discernible correlation with mitotic events or calcium signals could not be reliably detected (Tombes and Borisy, 1989). This confusion led to the view that, on balance, calcium was involved in mitosis but that the functionally relevant signals might be spatially restricted and/or temporally fleeting, rendering them difficult to reproducibly detect (Tsien and Tsien, 1990; Hepler, 1994). Sadly, this novel and potentially highly valuable area of research faded away around 15 years ago having failed to reach a consensus about the role of calcium during mitosis in mammalian cells.

## Recent Results

In the intervening period, many advances have been made in relevant areas of cell biology including microscopy, molecular biology, and calcium imaging. Perhaps one of the most useful innovations to have appeared in calcium signalling has been the development of a range of genetically encoded calcium indicators (GECIs). Pioneered in the laboratory of Roger Tsien, who developed the first cameleon FRET sensor calcium probes (Heim and Tsien, 1996; Miyawaki et al., 1997), but who, more importantly, forged the concept of constructing fluorescent calcium sensors using known properties of calcium binding proteins like calmodulin (CaM). This has since inspired others to develop non-FRET based sensors that have revolutionised the fields of calcium imaging in live cell systems (Akerboom et al., 2012; Akerboom et al., 2013; Chen et al., 2013; Yang et al., 2018). Taking an idea mentioned in a review article by Tsien, that the nature of mitotic calcium signals may make them difficult to capture reproducibly (Tsien and Tsien, 1990), and by applying next generation GECIs (developed as a result of his pioneering work into conceptualising these probes) mitotic calcium has been re-examined (Helassa et al., 2019). The idea of this work was to restrict the GECI to a unique cellular location (useful if the mitotic calcium signal is spatially constrained) and to use a probe with appropriate affinity (for detection of sub-micromolar magnitude signals) and calcium binding kinetics (so that transient calcium signals would be effectively reported). The chosen reporter, GCaMP6s (Chen et al., 2013), was targeted through fusion of the coding sequence to the cytoskeletal protein actin. Actin was selected as the targeting protein with the primary goal of identifying calcium signals at the contractile actin ring (Miller, 2011), the dense actin network that constricts the centre of the dividing cell during anaphase in readiness for cytokinesis. The probe failed to report contractile ring actin calcium but, unexpectedly, did detect two persistent calcium signals located at the spindle poles during metaphase and anaphase, which were subsequently identified, through colocalization, as the centrosomes of the dividing cell. This is interesting as historical studies reported calcium signals in the vicinity of the spindle poles however these observations were never confirmed, quantified, correlated, or investigated further (Silver, 1996). This calcium signal is functionally important as its removal with a focally activated calcium chelator inhibited mitosis progression. It is also noteworthy as recent research has highlighted centrosomes nucleate actin in mitotic cells possibly to control spindle microtubule dynamics (Farina et al., 2016; Farina et al., 2019). It seems plausible that these observations could be linked, and an exciting avenue of future investigation will be to understand how centrosomal calcium is generated and maintained and what downstream process(es) it controls. This work also observed, using transmission electron microscopy (TEM), ER close to centrosomes in mitotic cells, consistent with the historical studies examining IP_3_ signalling during mitosis as discussed above. A second recent study has also examined calcium and mitosis with specific focus on the role of IP_3_Rs in the orientation of the mitotic spindle (Lagos-Cabré et al., 2020). This work showed that the IP_3_R3 receptor subtype was required for correct spindle geometry, but that mitosis completed normally even when all IP_3_Rs were knocked-out or replaced by mutants, defective in gating calcium. It is known that cells lacking IP_3_Rs can successfully complete mitosis (Ando et al., 2018) and this latest paper indicates that calcium and IP_3_Rs have a relatively restricted role to play in ensuring the correct orientation of the spindle. This conservative effect on mitosis overall is at odds with the work on centrosomal calcium where suppression of the calcium signal blocked mitosis progression. This leads to the interesting possibility that the ER is not the only source of mitotic calcium and that additional mechanism(s) for generating mitosis specific calcium signals exist. Mitochondria have recently been reported to play a key role in cell division by providing ATP for the high-energy demands that this process requires. Energy production during mitosis is stimulated by a mitochondrial specific calcium signal that occurs globally during metaphase (Zhao et al., 2019) however it remains to be seen if mitochondrial calcium can influence non-mitochondrial mitosis specific processes. Indeed, from this report and a more recent, detailed characterisation of mitochondrial dynamics during mitosis (Moore et al., 2021), there is no obvious correlation between mitochondrial localisation and the spindle poles/mitotic spindles. Actin cables that are involved in the motion of mitochondria during mitosis (Moore et al., 2021) are excluded from the polar regions and therefore mitochondrial calcium signals would seemingly have to act ‘at a distance’ as it is unlikely, due to physical exclusion from the poles, that they could interact closely with factors controlling spindle organisation. Super-resolution confocal microscopy (SRCM) could be an interesting way to observe if a population of mitochondria do interact with the spindles through specific calcium signals or if a mitochondrial sub-population is able to access the polar regions through and alternative mechanism.

**FIGURE 1:**
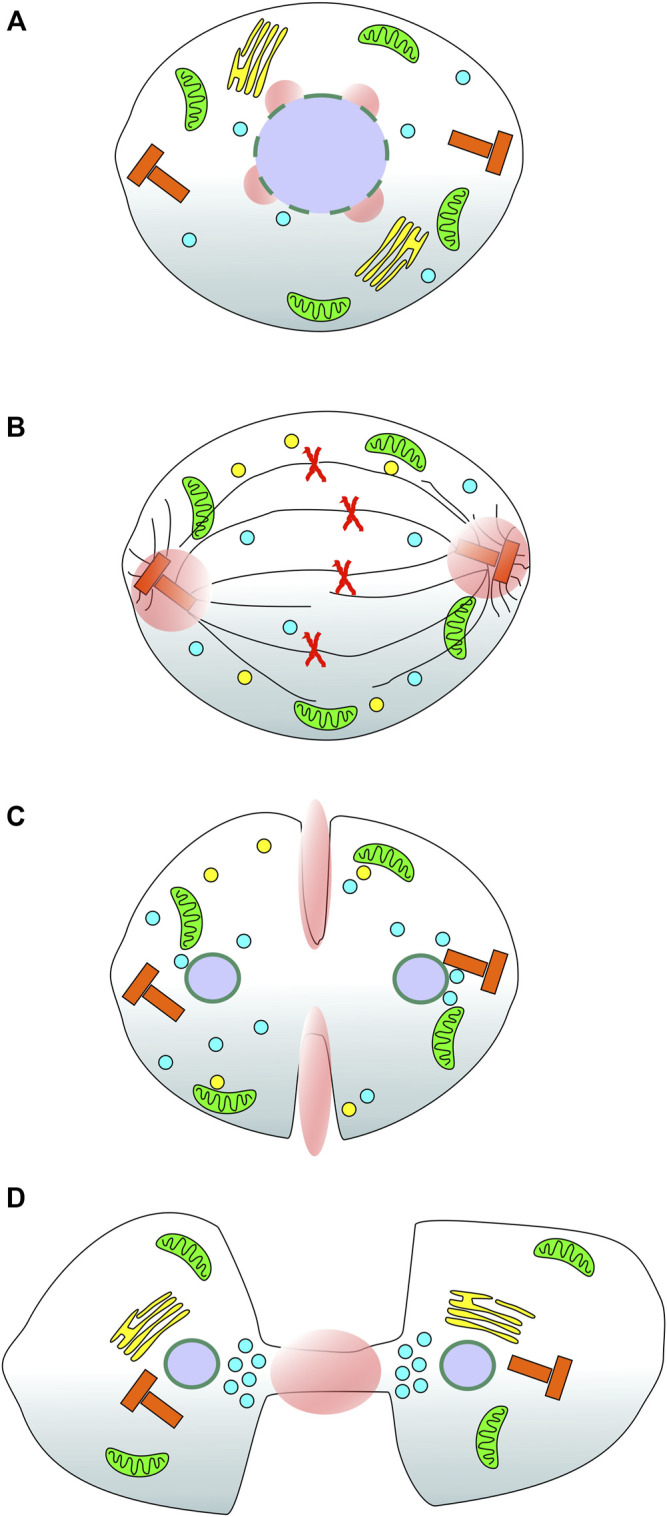
Reported and suspected sites of focal calcium signals during cell division in mammalian cells. A series of cartoons of a stylised mammalian cell as it progresses through mitosis. **(A)** During prometaphase, the nuclear envelope breaks down in response to a specific calcium signal (pink spheres); **(B)** During metaphase and into anaphase a focal calcium signal appears at both centrosomes of the dividing cell consistent with the dynamic movement of Annexin 11 from the nucleus to the spindle poles; **(C)** Based on the localisation of annexin A2 during mitosis, it is speculated that there is localised calcium present at the contractile actin ring (equatorial cortex) of the dividing cell; **(D)** At telophase, based on the localisation of Annexin 11 and the functional consequences of disrupting CaBP7, Sorcin and S100A6 function, it is speculated that there is a focal calcium signal active at the intercellular bridge/midbody. Green organelles: mitochondria; yellow stacks in A & D: Golgi complex; yellow spheres in B & C: Golgi derived vesicles; Red X’s in B: duplicated chromosomes; Black lines in B, spindle and astral microtubules; Blue spheres: Lysosomes; Red T-shaped organelles: centrosomes; Purple spheres: cell nuclei; Pink spheres and ellipses: localised calcium signals.

Calcium could also be provided from the extracellular environment however, a major route to calcium influx into cells, store operated calcium entry (SOCE), has been shown to be inhibited during mitosis (Smyth et al., 2009; Smyth and Putney, 2012; Yu et al., 2019). Evidence has been gathered from certain mammalian cell types showing that extracellular calcium is required for mitosis progression (Boynton et al., 1976; Hazelton et al., 1979; Whitfield et al., 1979; Tombes and Borisy, 1989) and there are alternative calcium entry channels including those of the TRP family (Samanta et al., 2018). A comprehensive analysis of calcium entry from outside the cell during mitosis has yet to be reported and this represents another interesting avenue of investigation. A useful probe in this regard would be a GCaMP anchored to the cytoplasmic face of the plasma membrane which could report local calcium entry events during cell division. It would be simple to construct such a reporter through fusion of the GECI to an appropriate targeting motif such as the acylation consensus sequence from LCK protein tyrosine kinase (Kabouridis et al., 1997). To generate focal calcium signals from an extracellular source would require selective opening of influx channels in restricted areas of the membrane. Conceptually, this could be achieved by localised recruitment of cytoplasmic channel modulator proteins or through spatial restriction of the calcium channels in the membrane. This type of event would perhaps most likely occur where the calcium signal is required close to the inner leaflet off the membrane otherwise the calcium would have diffuse some considerable distance to its target, enhancing the likelihood of off-target effects. Examining known and hypothesised sites of focal calcium during mitosis, Figure 1, it is possible to speculate that contractile ring and intercellular bridge calcium signals would be suited to control by extracellular calcium sources. More broadly, the targeting of GECIs to specific subcellular locations, in the context of this review, those important during mitosis, represents a novel way to map in detail specific calcium signalling events during cell division. The proof of principle for such an approach, reported by our laboratory (Helassa et al., 2019), opens the doorway to generating an atlas of cell-cycle specific calcium signals which can then be functionally investigated to assess their relative contributions and importance. We can say with some confidence that calcium plays a role at NEB, metaphase → anaphase transition and now, at centrosomes throughout metaphase and into anaphase (Figure 1). It is possible that other mitosis specific calcium signals exist and the approach of selectively targeting GECIs will help to test this idea.

### Calcium Signals Exist During Mitosis, What Are the Targets?

Calcium influences mitosis, so what are its molecular targets? The mitotic spindle has long been known to exhibit a sensitivity to calcium (Salmon and Segall, 1980; Izant, 1983) and one effect of disrupting IP_3_Rs is to cause misorientation of spindles (Lagos-Cabré et al., 2020) which may be due to inhibition of a key mitotic calcium signal. Calcium can exert cellular effects by directly interacting with target proteins or by binding to specific calcium sensor proteins which then convert the signal into a biological response (Burgoyne et al., 2019). The ubiquitous calcium sensor calmodulin accumulates at spindle poles, is associated with spindle microtubules during mitosis (Yu et al., 2004) and is required for mitosis progression (Rasmussen and Means, 1989; Stirling et al., 1996; Török et al., 1998; Li et al., 1999). It is therefore a strong candidate for mediating mitosis specific calcium driven events, including spindle behaviour, and its role in spindle orientation requires further investigation. Calmodulin is the primordial member of a large family of evolutionarily linked small EF-hand containing calcium sensor proteins (Burgoyne et al., 2019). Other members of this family have now been implicated in mitosis, most recently CaBP7 (also known as calneuron II, (McCue et al., 2009; McCue et al., 2010; Neumann et al., 2010; Rajamanoharan et al., 2015)), which, through its ability to regulate the phosphoinositide 4-kinase IIIβ enzyme, appears to influence a late stage of mitosis/cytokinesis (Rajamanoharan et al., 2015). This study revealed another potentially interesting facet of mitosis regulation involving lysosome function (Nugues et al., 2018) and which is linked, through CaBP7, to calcium. Although no direct evidence implicating lysosomal calcium in mitosis has been reported to date, a lysosome specific calcium channel, two-pore channel 2 (TPC2), was also identified in the same high-throughput screen for proteins essential in mitosis and cytokinesis which identified CaBP7 (Neumann et al., 2010). It is well established that lysosomes act as multi-functional calcium signalling platforms (Lloyd-Evans and Waller-Evans, 2020) and this represents an exciting avenue of future research. There has been growing interest in lysosomes as druggable targets to treat human disease (Tang et al., 2020) and a lysosome specific function during mitosis could represent a new approach to targeting cell proliferation (Geisslinger et al., 2020). In addition to calmodulin and CaBP7, a variety of other small calcium sensing proteins have been linked to mitosis progression. Sorcin, a small penta-EF hand calcium sensor, exhibits mitosis specific changes in cellular localisation (Lalioti et al., 2014) and is over-expressed in multiple human cancers (Zhou et al., 2019). It has been linked to the multidrug resistant phenotype of certain cancers and is therefore of significant interest as a clinically relevant target (Pomeroy et al., 2002; Tan et al., 2003; Yokota et al., 2006; Nagpal and Das, 2007; Zhou et al., 2019). Early in mitosis, sorcin-positive vesicles associate with the spindle and later, at cytokinesis, they are found in the cleavage furrow and at the midbody (Lalioti et al., 2014). Importantly, sorcin has been shown to physically associate with, and be phosphorylated by, polo-like kinase 1 (PLK1), a key regulator of mitosis (Lee et al., 2014). It induces PLK1 autophosphorylation and depletion of sorcin promotes cytokinesis failure and the appearance of multi-nucleate cells reminiscent of the phenotype observed with CaBP7 knockdown (Neumann et al., 2010). These loss-of-function observations from two independent small calcium sensing proteins suggests that cytokinesis is particularly sensitive to calcium. This is further consistent with a calcium dependent process such as exocytosis (Burgoyne and Morgan, 1993; Barclay et al., 2005) operating during cell scission (Goss and Toomre, 2008). Identification of specific focal calcium signals at the final stage of cell division will be an important future line of enquiry.

The S100 proteins are another family of conserved EF-hand containing calcium binding proteins with a wide range of physiological functions. S100A6 (also known as Calcyclin, (Donato et al., 2017)) is upregulated in proliferating cells (Nowotny et al., 2000) and is associated with the midbody during cell division (Skop et al., 2004). Disruption of S100A6 function causes defects in chromosome segregation (Ai and Skop, 2009) and inhibits cell proliferation (Breen and Tang, 2003) indicating that it has important functions during mitosis. S100 proteins interact with annexins to mediate recruitment to cellular membranes (Rintala-Dempsey et al., 2008) and this family of proteins have also been implicated in cell division.

The annexins are a large family of calcium dependent phospholipid binding proteins (Gerke et al., 2005) that are involved in a range of physiological processes including cell division. There are at least 12 different annexin proteins expressed in humans (Gerke and Moss, 2002) and some of these have been directly linked to roles in mitosis. Annexin 11 exhibits complex mitosis related trafficking first leaving the nucleus and associating with the spindle poles (consistent with our findings of a focal calcium signal at centrosomes) and later, accumulating at the intercellular bridge (Tomas et al., 2004). Importantly, depletion of annexin 11 inhibits midbody formation with daughter cells unable to complete cytokinesis (Tomas et al., 2004). A second member of the family, annexin A2, associates with the equatorial cortex during mitosis and its depletion, like annexin 11, inhibits cytokinesis albeit through a different mechanism that disrupts normal recruitment and function of the key GTPase RhoA thereby preventing normal contractile actin ring assembly (Benaud et al., 2015). As mentioned above, there is interplay between annexins and sorcins, and understanding this and how it links to localised calcium signals, that must be present to mediate the specific recruitment of these proteins, is a fertile area of future research.

## Discussion

An overarching theme of calcium regulation during mitosis is the use of focal signals to recruit specific calcium binding proteins which we then speculate execute mitosis and cytokinesis specific functions. There is much interesting research to complete in this area, first, a directed calcium sensing toolkit of localised probes to identify sites of focal calcium needs to be developed. The first such probe has revealed a centrosome calcium signal however there is good reason to believe that other focal signals exist, at the nucleus, equatorial cortex and midbody, based on historical observations of calcium signals and the localisation of calcium binding proteins involved in mitosis during cell division (Figure 1). The second is to understand what these signals control and how. This will require identification of the specific calcium binding proteins that respond to each focal signal and, in turn, what effectors they interact with to control downstream events. Understanding how calcium controls cell division is becoming increasingly important with more and more reports of dysregulated calcium signalling and aberrant expression of calcium binding proteins linked to human diseases including cancer. New insights in this area of cell biology could provide future targets for specific control of cell proliferation and lead to the development of new classes of anti-cancer drugs (Sansregret and Swanton, 2017).
